# Imperial Health Conference

**Published:** 1914-05-09

**Authors:** T. Nicolson

**Affiliations:** Organiser, Victoria League Imperial Health Conference and Exhibition. 6 Bloomsbury Square, London, W.C.


*To the Editor of* The Hospital.

### Imperial Health Conference.

*To the Editor of* The Hospital.

Sir,—Many of your readers who will be attending the May meetings will be interested in some of the practical steps in social reform going on in the Metropolis. Several will probably go to the Hampstead Garden Suburb, where the spires of the Established and Free Churches are the conspicuous landmarks in a Suburb that has set the pace for improved housing conditions throughout the land. They will be able to obtain information and guides at Temple Fortune House, Hampstead Way, and a post-card addressed to the Secretary there will ensure a welcome.

From May 18th to the 21st an Imperial Housing Conference and Exhibition will be held at the Imperial Institute. Provincial friends visiting London about that time will find much of interest there, and I shall be pleased to hear from ministers and representatives to the May meetings with a view to forming parties to make inspection of the models, plans, etc., under the guidance of experts. Free tickets of admission will also be available to all who send me their names and addresses.

Those interested in the housing problem will thus be provided with an armoury of facts and illustrations that will be of special value when speaking on or studying the question later in the year.—Yours faithfully,

T. Nicolson,
Organiser, Victoria League Imperial Health Conference and Exhibition.

6 Bloomsbury Square, London, W.C.

